# Development of a predictive model based on clinical indicators for refractory Mycoplasma pneumoniae pneumonia in children: a case–control study

**DOI:** 10.3389/fcimb.2026.1725669

**Published:** 2026-04-20

**Authors:** Feifei Yu, Yan Zhou, Zhengxiu Luo

**Affiliations:** 1 Department of Pharmacy, Children’s Hospital of Chongqing Medical University, National Clinical Research Center for Children and Adolescents’ Health and Diseases, Ministry of Education Key Laboratory of Child Development and Disorders, Chongqing Key Laboratory of Child Rare Diseases in Infection and Immunity, Chongqing, China; 2 Department of Respiratory Medicine, Children’s Hospital of Chongqing Medical University, National Clinical Research Center for Children and Adolescents’ Health and Diseases, Ministry of Education Key Laboratory of Child Development and Disorders, Chongqing Key Laboratory of Child Rare Diseases in Infection and Immunity, Chongqing, China

**Keywords:** children, indicators, predictive model, refractory Mycoplasma pneumoniae pneumonia, treatment

## Abstract

**Objective:**

This study aims to screen indicators for predicting the occurrence of refractory Mycoplasma pneumoniae pneumonia (RMPP) in children, determine the combined factors for predicting RMPP, and provide a basis for the early identification of children with RMPP and the determination of treatment plans.

**Methods:**

This study was a retrospective case–control analysis. A total of 522 children with MPP and 28 clinical indicators were included. Clinical feature, hospitalization period, laboratory data, etc., were collected. The risk factors related to RMPP were screened through univariate analysis. A multivariate logistic regression model was established, and stepwise regression was used to screen out independent risk factors. The operating characteristic curve (ROC) of the combined predictor was drawn for predictive efficacy analysis. A visual nomogram model for predicting the probability of RMPP occurrence was constructed and validated.

**Results:**

Differing from other results, there were no statistically significant differences in demographic indicators such as age and gender between the two groups. The multivariate logistic regression analysis showed that duration of fever (OR = 1.407), PLT (OR = 0.997), pleural effusion (OR = 2.084), atelectasis (OR = 3.116), and extrapulmonary complications (OR = 4.251) were independent risk factors for RMPP (P < 0.05). MP antibody titer ≥1:320 (OR = 0.420) is a protective factor. The AUC of the prediction model was 0.870 (95%CI: 0.837, 0.904), the sensitivity of the prediction model was 82.2%, the specificity was 80.5%, and the prediction accuracy was relatively high. The calibration curve, close to the 45° line, exhibited good calibration.

**Conclusion:**

We constructed and validated a visual and user-friendly model for individualized prediction of RMPP risk in children at initial presentation, to support clinical decision-making regarding macrolide therapy. This model provides a tool for identification of high-risk children, which may inform closer monitoring and prompt consideration of adjunctive therapies.

## 1 Introduction

Mycoplasma pneumoniae (MP) is an important pathogen causing respiratory tract infections in children, accounting for 20% to 40% of community-acquired pneumonia cases. Its incidence rate is higher during the epidemic season for older children (Wang et al., 2024). It is also usually endemic, with a major epidemic occurring every 4 to 7 years (Ding et al., 2024). Especially in the 2 years following the COVID-19 outbreak, the incidence of MPP has shown a clear upward trend in many countries. Since the beginning of 2023, Mycoplasma pneumoniae pneumonia (MPP) was widely prevalent throughout China (Yan et al., 2024). Macrolide antibacterial drugs remain the first choice for the treatment of MPP. However, with the gradual increase of MPP cases, cases of refractory Mycoplasma pneumoniae pneumonia (RMPP) are increasing, and clinicians often need to switch to infrequently used second-line agents, such as quinolones or tetracyclines (Tong et al., 2022; Dumke, 2024; Dungu et al., 2024).

At present, the pathogenesis of RMPP is not yet fully understood. It is generally believed to be related to factors such as excessive inflammatory response, MP resistance, abnormal secretion of airway mucus, and mixed infection. Meanwhile, macrolide-resistant M. pneumoniae (MRMP) infection is increasingly prevalent worldwide (Kim et al., 2022; Yang et al., 2025). This also poses a major challenge for clinical management. At present, it is believed that the mutations of MP macrolide resistance genes mainly occur at positions 2063 and 2064, which is in the 23 SrRNA domain V. The mutation at position 2063 causes high resistance in the 14- and 15-membered lactone rings, whereas the mutation at position 2064 mainly leads to high resistance in the 16-membered lactone ring. In fact, we found that the detection of the drug resistance gene has been applied to most MPP patients with a tendency toward severe illness, identifying drug resistance for switching to second-line treatment in clinical practice, although there is no unified conclusion (Chen et al., 2020; Choi et al., 2022). However, it should be noted that the genetic mutation, such as the A2063/2064G mutation, does not always match the clinical efficacy and is not the only indicator to changing the treatment (Cheng et al., 2024; Cheng et al., 2025; Shen et al., 2025).

Compared with ordinary MPP/macrolide-unresponsive Mycoplasma pneumoniae pneumonia (MUMPP), RMPP has a longer duration of fever and hospital stay, increased dosage of anti-infective drugs, and higher risk of recurrent respiratory tract infections. It often leads to atelectasis, lung necrosis, bronchiectasis, and obliterative bronchiolitis and is more prone to extrapulmonary complications. A few children’s conditions, although not severe, have persistent symptoms such as coughing and shortness of breath, presenting as chronic pneumonia, secondary respiratory hyperreactivity leading to bronchial asthma, etc (Zhai et al., 2020; Xu et al., 2024). Compared with MPP, RMPP has similar initial symptoms, laboratory test results, and imaging manifestations. Delayed effective antibiotic treatment is associated with prolonged and/or more severe disease (Tsai et al., 2021).

There is a lack of sensitive markers for assessing the severity and prognosis of the disease. As our understanding of MPP has deepened in recent years, many biomarkers have been considered that are associated with the early recognition, determination of severity, and prognostic assessment of RMPP. Moreover, with the advancement of Internet-based information technology, a range of predictive models has emerged. Nevertheless, we have also identified some shortcomings. Firstly, most current research is limited to single-center retrospective studies with a narrow sample scope and limited sample size. Some studies only include a few dozen of cases, leading to diagnostic cutoff values that may not have good universality. Secondly, some studies have different inclusion criteria for RMPP and may actually include MUMPP cases. Moreover, the indicators included in some predictive models are not common laboratory/examination data or are more subjective indicators (e.g., serum uric acid, lung ultrasound, consolidation size/BSA, and radiological imaging change) in the general clinical process of pneumonia, which poses certain obstacles to clinical application. Finally, after comparing these studies, we found that some studies collected samples before the COVID-19 pandemic, and their representativism and coverage of post-COVID-19 cases are weaker (Liu et al., 2022; Xie et al., 2022; Li et al., 2023; Ding and Jiang, 2025).

To ensure representativeness, we meticulously screened patients admitted to the respiratory department of a tertiary children’s hospital, which served as a regional medical center. The selected timeframe was the 3-year period following the onset of the COVID-19 outbreak in China. Moreover, to guarantee operability, 28 common clinical indicators were collected to develop a risk prediction model for children with refractory mycoplasma pneumonia (RMPP). This model holds significant importance as it enables healthcare professionals to recognize high-risk children at an early stage, formulate timely treatment strategies, mitigate extrapulmonary complications, and enhance outcomes (Jiang et al., 2025). This study intends to offer a reference for identifying predictors of RMPP in hospitalized children in China.

## 2 Methods

This study was an observational one. A total of 2,020 children with MPP were admitted to the Department of Respiratory Medicine of Children’s Hospital Affiliated to Chongqing Medical University from January 1, 2020, to December 31, 2023. MPP can be diagnosed as follows: (1) presence of fever, cough, tachypnea, difficulty in breathing; (2) chest X-ray or CT examination results compatible with pneumonia; and (3) detection of MP IgM antibody conducted using enzyme-linked immunosorbent assay during the illness. Either a single serum MP antibody titer ≥1:160 or positive MP-DNA PCR was required for diagnosis. RMPP was documented as persistent fever, aggravated clinical signs, and progressive imaging findings despite the administration of macrolide for 7 days or more. The remaining children were NRMPP defined as the reduction of fever and improvement in clinical signs or imaging findings within 7 days of macrolide (azithromycin) treatment (National Health Commission of the People’s Republic of China, 2023).

**Figure 1:**
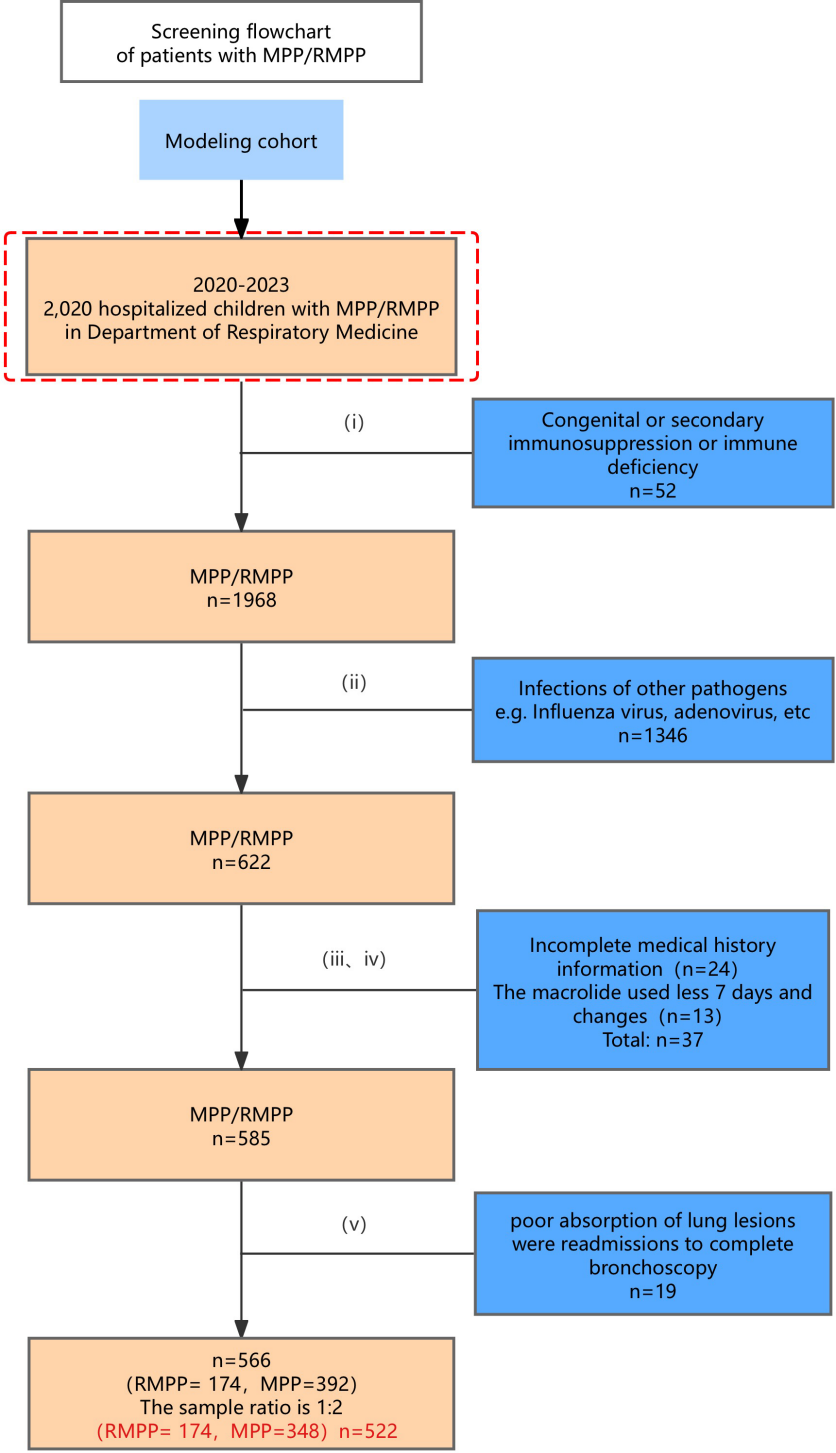
Screening flowchart of patients with MPP/RMPP in the modeling cohort.

The inclusion criteria were as follows: (i) Patients were under the age of 18 years, (ii) had MPP, (iii) were confirmed as MUMPP, and (iv) continued to be treated with macrolide (azithromycin) after the confirmation of MUMPP to 7 days without antibiotic changes. The exclusion criteria were as follows: (i) congenital or secondary immunosuppression or immune deficiency (congenital immunodeficiency, immunosuppressive therapy, or HIV infection); (ii) clear coinfection with other pathogens, which were identified by microbiological culture, PCR, or serology for other pathogens; (iii) RMPP was diagnosed despite the use of macrolide (azithromycin) for less than 7 days; (iv) incomplete medical history information; (v) poor absorption of lung lesions led to readmissions for complete bronchoscopy. According to twice the number of cases in the RMPP group (174 children), 348 children with NRMPP were selected as the MPP group by simple random sampling. A flowchart of patient enrollment is shown in **Figure 1**.

This study was approved by the Medical Ethics Committee of the Children’s Hospital Affiliated to Chongqing Medical University (Approval No. (2023) Lun Shen (Yan) No. (285)). The requirement for written informed consent was waived due to the retrospective nature of the study. This waiver was in accordance with the hospital’s retrospective research policy and the ethical guidelines of the Declaration of Helsinki (1964 and subsequent revisions). This study did not involve clinical trials or animal experiments, and all data processing strictly complied with the above ethical standards.

### 2.1 Data collection

In order to ensure the consistency of diagnosis and treatment of MPP/RMPP patients, these patients were admitted to the Department of Respiratory Medicine of Children’s Hospital Affiliated to Chongqing Medical University, from January 1, 2020, to December 31, 2023. A total of 28 clinical indicators included clinical features (age, sex, and weight), hospitalization period, treatments, fever peak, duration of fever, and clinical manifestations on admission such as chills, rash, cough, chest pain, and wheezing. Among them, duration of fever was defined as the total number of days with fever (axillary temperature ≥37.5 °C) reported from symptom onset to the time of initial assessment and data collection at presentation, which was the time of hospital admission. Within 24 h after admission, patients in both the RMPP and the MPP underwent tests for white blood cell count (WBC), neutrophilic granulocyte percentage (N%), percentage of lymphocytes (L%), hemoglobin (HB), C-reactive protein (CRP), procalcitonin (PCT), lactate dehydrogenase (LDH), blood platelet count (PLT), fibrinogen (FIB), and the first imaging findings were collected whether there is pleural effusion (unilateral/bilateral), lung consolidation, atelectasis, lung necrosis, etc. According to the Peroni criteria (Peroni and Boner, 2000), atelectasis is defined as decreased lung volumes with loss of air bronchograms, whereas consolidation is defined as alveolar filling with exudate but preserved volume. Chest X-ray or CT reports were independently reviewed by two pediatric radiologists who were unaware of clinical information (double-blind design). Extrapulmonary complications were diagnosed based on standard clinical, laboratory, or imaging criteria (e.g., rash, hepatitis, neurological symptoms). When the results were inconsistent, senior physicians other than the first two made a decision after discussion to minimize diagnostic bias.

### 2.2 Statistical analysis

Statistical analysis was conducted using R 4.2.3 software. The normal distribution is expressed as mean ± standard deviation (x ± s). Measurement data with non-normal distribution were expressed as M (Q_1_, Q_3_), and the Wilcoxon rank-sum test with two independent samples was used for comparison between the two groups. Categorical data were expressed as n (%). The comparison between the two groups was conducted using the χ^2^ test or the corrected χ^2^ test. We checked for multicollinearity using variance inflation factors (VIF), where a VIF >10 was considered highly collinear. All final models had low VIF (i.e., <5), suggesting a lack of collinearity among the variables. Multivariate logistic regression analysis was conducted using stepwise regression to screen independent predictors of RMPP occurrence and construct a visual nomogram model for predicting the probability of RMPP occurrence. The predictive performance of the model was evaluated using the receiver operating characteristic (ROC) curve. For the ROC curve analysis, the outcome variable (RMPP) was coded as a binary variable, with RMPP = 1 and non-RMPP = 0. The default ROC analysis direction was set such that continuous variables with higher values were initially assumed to be associated with a higher probability of the outcome (RMPP = 1). For predictors that demonstrated an inverse association with the outcome (i.e., a raw area under the curve [AUC]<0.5), the AUC was transformed by calculating 1-AUC to ensure all reported values are ≥0.5 and directly comparable. Model calibration was assessed via the calibration curve, and overall predictive accuracy was quantified using the Brier score (where lower scores indicate better accuracy). All statistical tests were two-sided, and P values < 0.05 were considered statistically significant.

## 3 Results

### 3.1 Comparison of clinical data and laboratory indicators between the two groups of children

A total of 2,020 MPP patients within the study period were screened; of those, 522 patients were included in this study. A total of 174 children were in the RMPP group, 348 were in the MPP group, 28 clinical indicators were collected; all data were actually observed and have been verified by two researchers. As shown in **Table 1**, there were no statistically significant differences between the RMPP and MPP groups in demographic indicators such as age (P = 0.073) and gender (P = 0.264). Weight showed a statistically significant difference (P = 0.042). We acknowledge that the clinical relevance of this small absolute difference in weight (23.19 vs. 21.59 kg) may be limited, and it was not selected as a predictor in the final multivariable model. The positive mutation rates of the drug resistance genes A2063G and A2064G in the RMPP group and the MPP group were 86.1% and 85.7%, respectively. The occurrence of pleural effusion (35.1%) and extrapulmonary complications (46.6%) in the RMPP group were significantly higher than those in the MPP group (9.2%, 12.4%), and the differences were statistically significant (P<0.001). There were statistically significant differences in the duration of fever, fever peak, PLT, N%, L%, CRP, PCT, LDH, hospital stay, and atelectasis that occurred in the RMPP group (P<0.001).

**Table 1:** Clinical characteristics and laboratory findings of MPP and RMPP patients.

Characteristics	RMPP (N=174)	MPP (N=348)	χ^2^/t/Z	P value
Demographic data				
Age (years), mean ± SD	6.68 ± 2.54	6.24 ± 2.61	1.798	0.073
Gender			1.245	0.264
Male (n,%)	76 (43.7)	170 (48.9)		
Female (n,%)	98 (56.3)	178 (51.1)		
Weight (kg), mean ± SD	23.19 ± 9.22	21.59 ± 8.05	2.035	0.042
Clinical manifestation				
Chills	70 (40.2)	124 (35.6)	1.050	0.305
Rash	10 (5.7)	16 (4.6)	0.324	0.569
Cough (days)	173 (99.4)	347 (99.7)	0.251	0.616
Chest pain	4 (2.3)	2 (0.6)	3.035	0.081
Wheezing	14 (8.0)	59 (17.0)	7.652	0.006
Fever peak (°C)	39.8 (39.2, 40.0)	39.3(39.0, 39.8)	6.391	<0.001
Duration of fever (days)	12.00 (10.00, 14.00)	7.50 (6.00, 9.25)	12.166	<0.001
Laboratory tests				
WBC (×10^9^/L)	7.30 (5.56,9.73)	7.03 (5.66,9.36)	0.382	0.702
PLT (×10^9^/L)	274.5 (219.0, 343.8)	318.5 (252.8, 389.0)	4.139	<0.001
N,%	66.9 (58.5, 75.0)	62.2 (53.9, 69.4)	5.185	<0.001
L,%	24.6 (17.8, 34.5)	29.5 (23.2, 37.1)	5.182	<0.001
HB (g/L)	118.0 (111.0, 127.0)	121.0 (114.0, 127.0)	1.841	0.066
CRP (mg/L)	16.05 (8.00, 31.07)	9.35 (5.43, 20.11)	5.500	<0.001
PCT (ng/ml)	0.14 (0.09, 0.37)	0.09 (0.06, 0.15)	7.570	<0.001
FIB (g/L)	4.53 (3.90, 5.10)	4.34 (3.77, 4.92)	2.752	0.006
LDH (U/L)	321.5 (270.3, 450.8)	283.0 (251.8, 328.3)	5.846	<0.001
Hospital stay (days)	7.0 (6.0, 9.0)	6.0 (5.0, 7.0)	9.502	<0.001
MP antibody titer (≥1:320)	134 (77.0)	302 (86.8)	8.047	0.005
Macrolide resistance gene mutation (A2063G/A2064G), n (%)	86.1% (112/130)	85.7% (126/147)		0.916
Imaging findings				
Lung consolidation (n, %)	116 (66.7)	177 (50.9)	11.767	0.001
Cavitary shadow (n, %)	3 (1.7)	2 (0.6)	1.615	0.204
Pleural effusion (n, %)	61 (35.1)	32 (9.2)	52.989	<0.001
One-sided (n, %)	46 (26.4)	26 (7.5)		
Two-sided (n, %)	15 (8.6)	6 (1.7)		
Atelectasis (n, %)	40 (23.0)	23 (6.6)	29.325	<0.001
Pulmonary necrosis (n, %)	5 (2.9)	1 (0.3)	6.828	0.009
Other				
Extrapulmonary complications, (n, %)	81 (46.6)	43 (12.4)	74.891	<0.001

### 3.2 Multivariate logistic regression analysis of the influencing factors of RMPP occurrence

The dependent variable was the occurrence of RMPP (no=0, yes=1). Independent variables with statistical significance in univariate analysis included: duration of fever (measured value), peak fever temperature (measured value), platelet count (PLT, measured value), neutrophil percentage (N%, measured value), lymphocyte percentage (L%, measured value), C-reactive protein (CRP, measured value), procalcitonin (PCT, measured value), MP antibody titer ≥1:320 (no=0, yes=1), fibrinogen (FIB, measured value), lactate dehydrogenase (LDH, measured value), pleural effusion (absent=0, present=1), extrapulmonary complications (absent=0, present=1), pulmonary consolidation (absent=0, present=1), atelectasis (absent=0, present=1), and pulmonary necrosis (absent=0, present=1). Forward stepwise regression is an automated and systematic approach to variable selection. This allows us to efficiently identify the most relevant predictors from a large set of potential variables, reducing the risk of including irrelevant or redundant factors. Through this, we obtain a parsimonious model that is easier to interpret. Forward conditional stepwise logistic regression analysis identified the following independent risk factors for RMPP: extrapulmonary complications (OR = 4.251), PLT (OR = 0.997), atelectasis (OR = 3.116), pleural effusion (OR = 2.084), and duration of fever (OR = 1.407) (all P<0.05). MP antibody titer MP≥1:320 (OR = 0.420) was identified as a protective factor against RMPP (P<0.05). The results are presented in **Table 2**.

**Table 2:** Multivariate logistic regression analysis of RMPP occurrence (forward condition method).

Indexes	B	S.E	Wald	P value	OR	95% CI
Duration of fever	0.342	0.042	64.924	0.000	1.407	1.295~1.529
PLT	−0.003	0.001	5.199	0.023	0.997	0.995~1.000
Pleural effusion	0.734	0.302	5.899	0.015	2.084	1.152~3.769
Atelectasis	1.136	0.366	9.628	0.002	3.116	1.520~6.387
Extrapulmonary complications	1.447	0.278	27.020	0.000	4.251	2.463~7.337
MP antibody titer ≥1:320	−0.868	0.308	7.945	0.005	0.420	0.230 ~0.768

### 3.3 Construction of a nomogram for identification of RMPP

**Figure 2:**
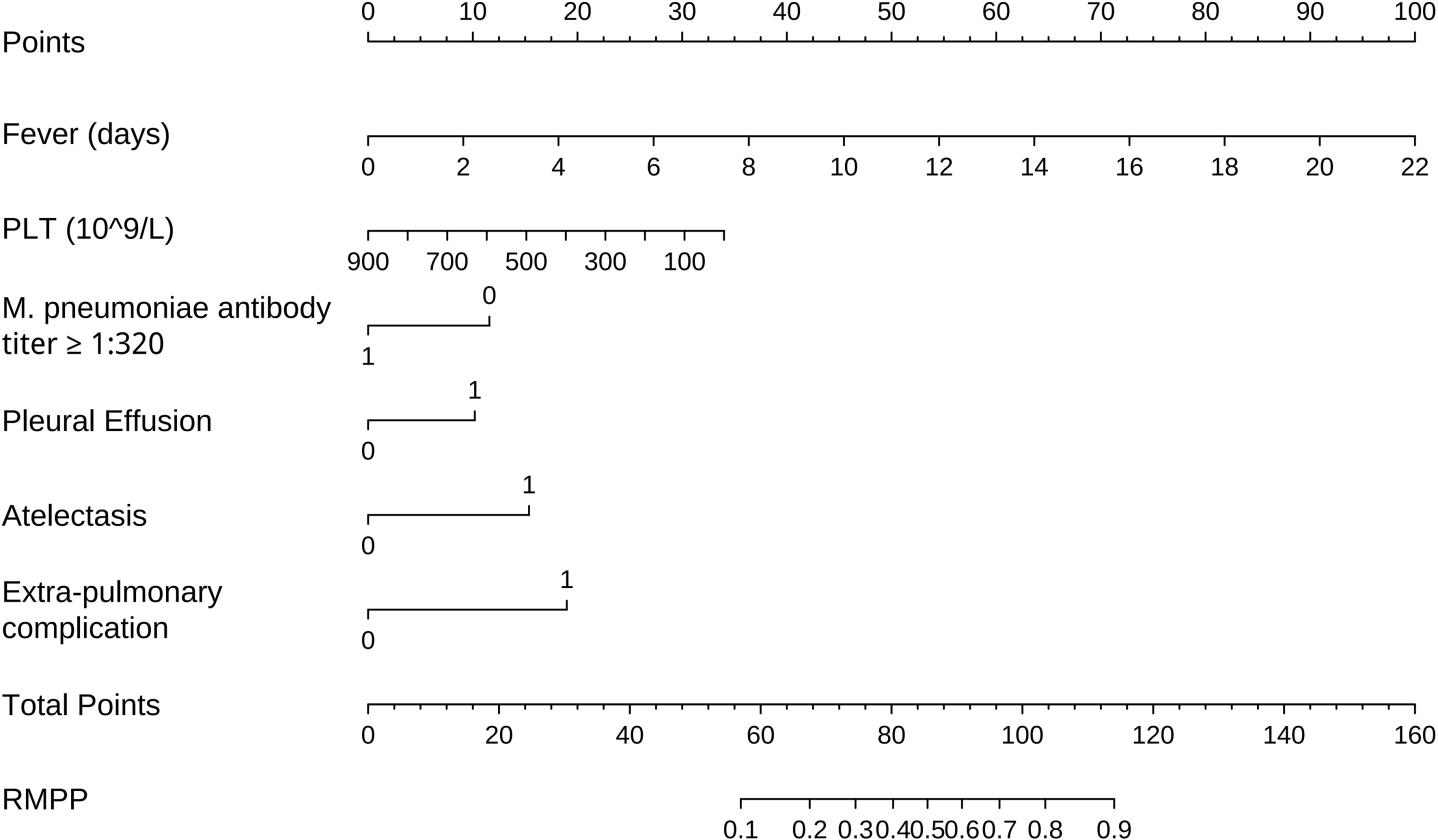
Nomogram for predicting RMPP in child patients. RMPP, refractory Mycoplasma pneumoniae pneumonia; PLT, blood platelet count.

Based on multivariate logistic regression analysis, five risk factors (duration of fever, PLT, pleural effusion, atelectasis, extrapulmonary complications) and one protective factor (MP antibody titer ≥1:320) were screened out. A visual nomogram for predicting the probability of RMPP occurrence was drawn, as shown in **Figure 2**. An example for a better illustration is a 4-year-old patient who had 12 days of fever, 318×10^9^/L of PLT, MP antibody titer ≥1:320, no pleural effusion, atelectasis, no extrapulmonary complications, and a total point of 105 (55 + 23 + 12 + 0 + 15 + 0). The risk was 0.8. This child patient eventually developed severe RMPP and was hospitalized four times due to severe RMPP.

### 3.4 Validation of a nomogram for identification of RMPP

**Figure 3:**
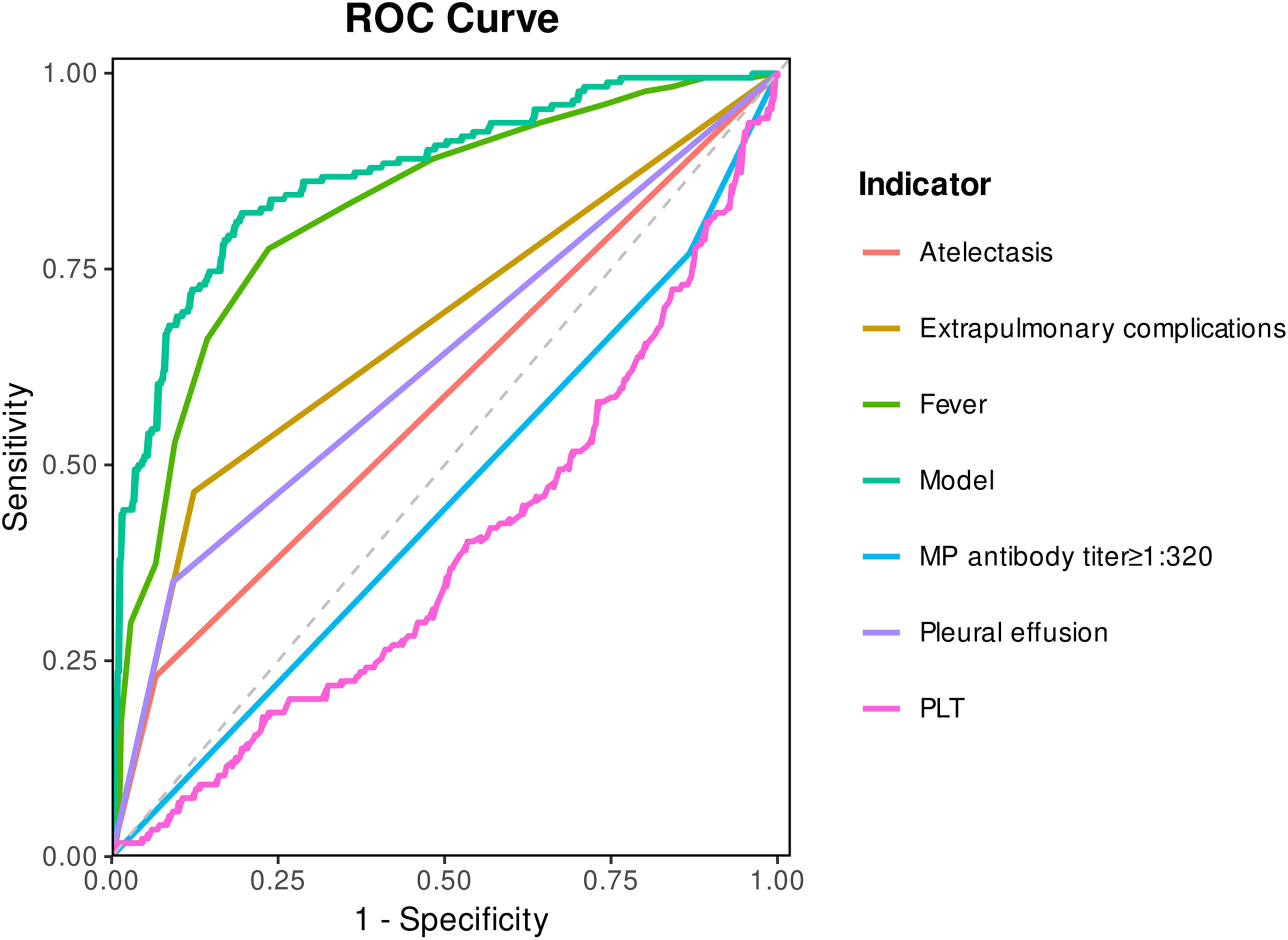
The ROC analysis for the predictive model. ROC, receiver operating characteristic.

**Figure 4:**
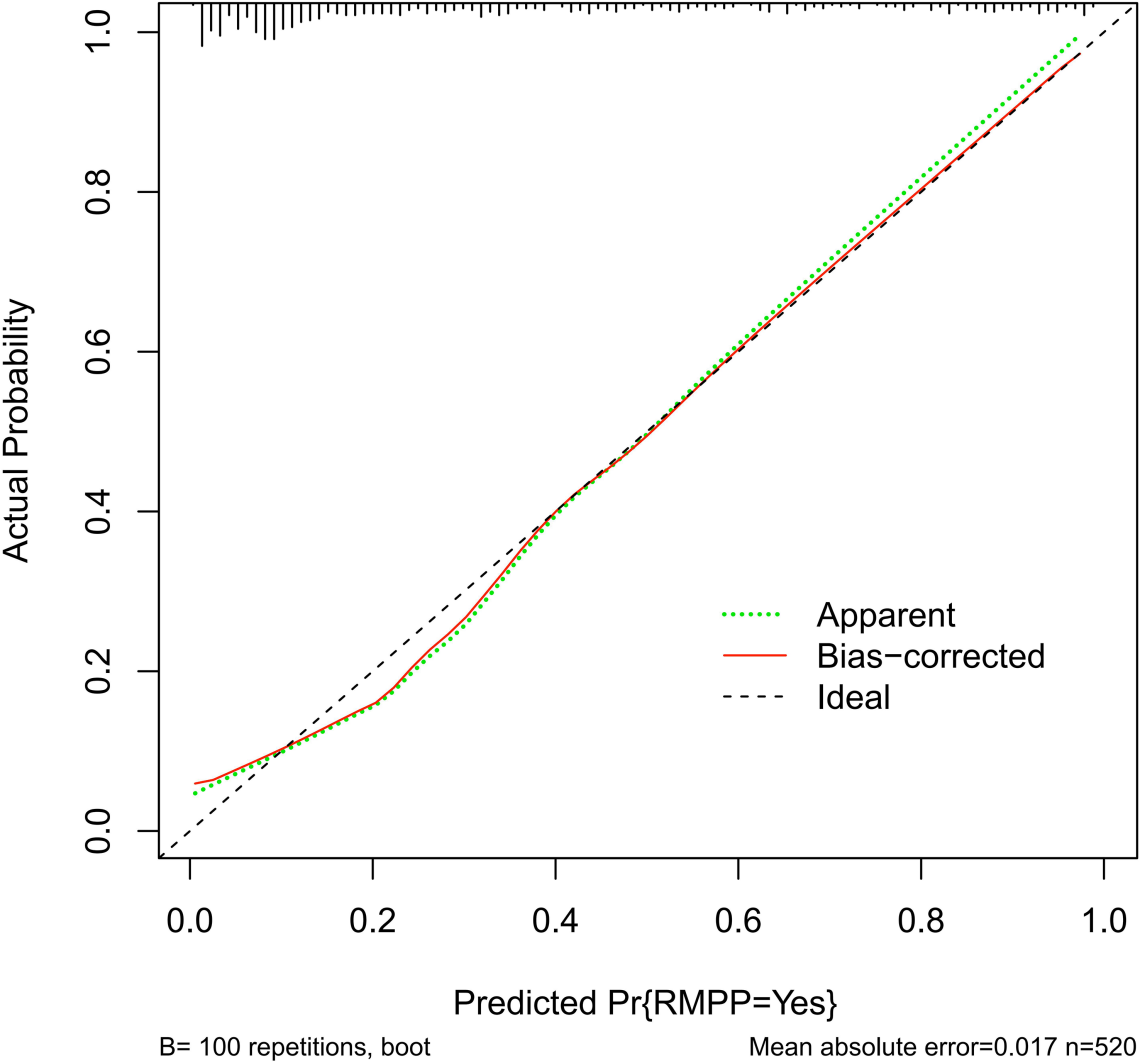
The calibration curve indicated good consistency between the actual diagnosed RMPP and the predicted probability. RMPP, refractory Mycoplasma pneumoniae pneumonia.

The ROC curves of each independent variable and the model are shown in **Figure 3**, and the area under the curve (AUC) of each variable is shown in **Table 3**. The critical value of the duration of fever is 9.5 days, and the critical value of PLT is 264.5×10^9^/L. For single-variable prediction, the items with relatively large AUC were the duration of fever (AUC = 0.825), extrapulmonary complications (AUC = 0.671), and pleural effusion (AUC = 0.629), with sensitivities of 77.6%, 46.6%, and 35.1% respectively, and specificities of 76.4%, 87.6%, and 90.8% respectively. The AUC of the prediction model was 0.870 (95%CI: 0.837, 0.904), the sensitivity of the prediction model was 82.2%, and the specificity was 80.5%, indicating that the prediction accuracy of this model was relatively high. The calibration curve, close to the 45° line, exhibited good calibration of the nomogram (**Figure 4**). We also performed a rigorous sensitivity analysis by restricting the cohort to patients with a fever duration of less than 7 days at admission (n=199). As shown in the **Table 4**, when we excluded patients with longer pre-admission fever (i.e., those with a higher risk of information leakage), most individual predictors lost their statistical significance. However, critically, our multivariate prediction model maintained robust discriminative ability, with an AUC of 0.717 (95% CI: 0.611, 0.824), P = 0.002.

**Table 3:** Evaluation of the area under the ROC curve and the prediction effect.

Indexes	AUC (95CI)	P value	Sensitivity (%)	Specificity (%)
Pleural effusion (+)	0.629 (0.576, 0.683)	<0.001	35.1	90.8
Duration of fever (critical value 9.5 days)	0.825 (0.787, 0.864)	<0.001	77.6	76.4
PLT (critical value 264.5×10^9^/L)	0.611 (0.559, 0.663)	<0.001	52.9	27.9
Extrapulmonary complications (+)	0.671 (0.619, 0.723)	<0.001	46.6	87.6
Atelectasis (+)	0.582 (0.528, 0.636)	0.003	23.0	93.4
MP antibody titer≥1:320	0.549 (0.495, 0.602)	0.050	77.0	13.2
Model prediction	0.870 (0.837, 0.904)	<0.001	82.2	80.5

AUC values for PLT and MP antibody titer have been direction-adjusted (1-AUC) to reflect their positive association with RMPP risk after considering the low-value direction.

**Table 4:** Sensitivity analysis (restricting to patients with fever duration <7 days).

Factor	AUC (95% CI)	P value
Pleural effusion	0.493 (0.358, 0.628)	0.920
Duration of fever	0.599 (0.475, 0.723)	0.157
PLT	0.654 (0.564, 0.745)	0.027
Extrapulmonary complications	0.591 (0.446, 0.737)	0.191
Atelectasis	0.547 (0.404, 0.690)	0.501
MP antibody titer≥1:320	0.528 (0.386, 0.669)	0.692
Model prediction	0.717 (0.611, 0.824)	0.002

## 4 Discussion

MP infection is usually a benign and self-limited disease. Most children with MPP have a good prognosis after treatment with macrolide antibiotics, but in some cases, it can progress to refractory or severe, even life-threatening pneumonia. In recent years, the incidence of MPP in China has shown an upward trend; especially since the COVID-19 outbreak, when regional outbreaks of MPP occurred, the incidence of RMPP has shown an upward trend. RMPP has a long course of disease, more severe clinical manifestations, and a variety of complications.

Although the effect of switching to tetracyclines or quinolones for children with MUMPP is considerable, the clinical efficacy of macrolide (azithromycin) combined with immunoglobulin or glucocorticoids is also considerable. Therefore, it seems more important to find a more appropriate time to replace anti-infective drugs and avoid the emergence of antimicrobial drug resistance due to the overuse of second-line treatment drugs (tetracyclines or quinolones). It can also avoid the safety uncertainty of tetracyclines or quinolones, delayed diagnosis, delayed effective treatment, and even irreversible damage to children, so effective identification is more important.

In order to ensure the consistency of diagnosis and treatment of MPP patients, these patients were collected from the Department of Respiratory Medicine; 522 children were enrolled. A total of 28 clinical indicators were collected, including demographic data, clinical characteristics, imaging, blood cell analysis, and bronchoscopy results of the children were compared, analyzing the high-risk factors for the formation of RMPP. In this study, we found that the RMPP group had statistically significant differences in the duration of fever, fever peak, PLT, N%, L%, CRP, PCT, LDH, length of hospital stay, and the occurrence of pleural effusion, atelectasis, and extrapulmonary complications. The RMPP group was more likely to have extrapulmonary complications such as infectious erythema, liver function damage, coagulation dysfunction, and toxic encephalopathy, accounting for 46.6%. There was no significant difference in the positive rate of drug resistance genes between the two groups (P = 0.916), which may indicate that the single indicator of A2063/2064G site mutations are positive and may not accurately guide early drug treatment and decision-making drug treatment plan.

By analyzing the risk factors of RMPP, an easy-to-use prediction model for RMPP was established, hoping to provide support for identification of RMPP and guide clinical treatment. Through multivariate logistic regression analysis, a total of five indicators were screened: The duration of fever (OR = 1.407), PLT (OR = 0.997), pleural effusion (OR = 2.084), atelectasis (OR = 3.116), and extrapulmonary complications (OR = 4.251) were independent risk factors for RMPP (P<0.05). PLT and MP antibody titer are inversely associated with RMPP risk. After direction adjustment, a low platelet count (≤ 264.5×10^9^/L) was significantly associated with an increased risk of RMPP, with an AUC of 0.611 (95%CI: 0.559, 0.663). Moreover, the MP antibody titer ≥1:320 (AUC = 0.549, 95%CI: 0.495, 0.602) was a protective factor for RMPP (P < 0.05). The observed “protective” effect of the MP antibody titer may be heavily influenced by the timing of blood sample collection relative to disease onset. This temporal factor is a potential source of bias that cannot be fully excluded. Among them is prolonged fever as a marker of persistent inflammation. Low PLTs possibly reflect consumption or immune-mediated processes. PLTs play a supporting role in fighting infection as they contain pattern recognition receptors (PRRs) that detect pathogens. Binding of the ligand to the receptor leads to platelet activation and secretion of alpha and beta interferons, and interleukins including IL-6, which result in inhibition of platelet production by megakaryocytes. In addition, hyperactive platelets are recognized by the immune system and degraded (Kosidło et al., 2023). At the same time, a “cytokine storm” is observed in patients with a severe condition, in the course of which there is an abundant secretion of, among others, IL-6 and IL-8. Accordingly, a decrease in PLT level is observed due to sensitivity of megakaryocytes to signals that inhibit platelet formation (Delshad et al., 2021; Rohlfing et al., 2021). This suggests that RMPP may be accompanied by thrombocytopenia.

With imaging findings indicating disease severity, atelectasis causes local hypoxia in the lung tissue, increases the permeability of the alveolar barrier, and activates alveolar macrophages to release inflammatory mediators, which leads to edema in the lung tissue (Lee et al., 2021). Second, atelectasis leads to obstruction of bronchial drainage and mucus plugs and pathogens accumulate locally (Peroni and Boner, 2000), and the affected lung tissue of atelectasis has a reduced ability to clear pathogens (Drinkwater et al., 1981). In summary, this sustained hyperinflammatory response may lead to persistent fever and recurrent infections, resulting in further damage to the elastic fibers and smooth muscle of the bronchial wall. This compromises airway self-cleaning capacity, exacerbates pulmonary imaging abnormalities and clinical manifestations, and contributes to systemic involvement in the form of extrapulmonary complications. These interconnected processes establish a vicious cycle that ultimately predisposes children with MPP to the development of RMPP following macrolide treatment.

The AUC of the multivariate prediction model was 0.870 (95% CI: 0.837, 0.904), the sensitivity of the prediction model was 82.2%, and the specificity was 80.5%, indicating that the prediction accuracy of this model was relatively high. The calibration curve indicated good consistency between the actual diagnosed RMPP and the predicted probability. Apart from this, we performed a rigorous sensitivity analysis by restricting the cohort to patients with a fever duration of less than 7 days at admission (n = 199). Our multivariate prediction model maintained robust discriminative ability, with an AUC of 0.717 (95% CI: 0.611, 0.824), P = 0.002. It confirms that whereas individual risk factors may have limited predictive power in the very early stages of the disease, the combination of factors in our model provides a reliable assessment even for patients with shorter fever duration. This model can be applied to early hospitalization assessment to guide monitoring and potential therapy escalation.

### 4.1 Strengths of this study

At present, the exact pathogenesis of RMPP has not been fully elucidated. The initial symptoms of RMPP are similar to those of MPP, which makes the clinical treatment of RMPP less predictable and often delays use of effective drugs. On the other hand, premature change to tetracyclines or quinolones also appears to have some negative effects. The development of a method to find a better time to replace anti-infective drugs is required.

After the global COVID-19 pandemic in 2020, MPP had a large-scale outbreak in China in 2022, and our cases were specifically selected from this timeframe. In addition to that, to ensure homogeneity in diagnostic and therapeutic management, all enrolled patients were recruited from the respiratory department of our institution. Some studies (Wen et al., 2021; Huang et al., 2022; Liu et al., 2022; Xie et al., 2022; Li et al., 2023; Ding and Jiang, 2025) have constructed a model for identification of RMPP in children, but not based on the context of this era. Moreover, some indicators selected by prediction models are not common laboratory/examination data in the routine diagnosis and treatment of pneumonia, or may be more subjective (such as serum uric acid, lung ultrasound, consolidation area/body surface area ratio, and imaging changes), which also bring certain obstacles to clinical practical application (Liu et al., 2022; Xie et al., 2022; Li et al., 2023; Ding and Jiang, 2025). We constructed and validated a visual and user-friendly model for individualized prediction of RMPP risk in children at initial presentation.

### 4.2 Limitations of our study

As this study is a retrospective single-center study, the analysis with existing data may lead to incomplete data; thus, there is a risk of incomplete predictors or neglect due to missing data. In addition, retrospective studies were conducted after the outcome had occurred, making the causal relationship of predictors to the outcome uncertain. The use of forward stepwise regression is a pragmatic approach to identify a parsimonious set of predictors in a clinical setting, and we also acknowledge its limitations (e.g., potential overfitting, instability). In this study, parameters such as D-dimer, MP-DNA load in bronchoalveolar lavage fluid, and cytokine levels were not effectively included in the analysis due to missing data. The practicability of the prediction model may need to be external validation in the further, such as validation using multicenter samples.

## 5 Conclusions

In summary, the combination of the duration of fever, PLT, pleural effusion, atelectasis, and extrapulmonary complications (such as infectious erythema, liver function damage, coagulation dysfunction, and toxic encephalopathy) were independent risk factors for RMPP. We constructed and validated a visual and user-friendly model for individualized prediction of RMPP risk in children at initial presentation, to support clinical decision-making regarding macrolide therapy. According to the nomogram model, continuation of macrolide should be considered rather than second-line antibiotics including tetracyclines (doxycycline or minocycline) and fluoroquinolones for MUMPP children with low predictive values. This model provides a tool for identification of high-risk children, which may inform closer monitoring and prompt consideration of adjunctive therapies.

## Data Availability

The original contributions presented in the study are included in the article/supplementary material. Further inquiries can be directed to the corresponding author.
